# A Hospital for the Application of the Blood Treatment—Haematherapy

**Published:** 1897-12

**Authors:** 


**A Hospital for the Application of the Blood Treatment —Haematherapy**—has been established at Stamford, Connecticut, and is under the charge of Dr. T. J. Biggs. Dr. Biggs has had quite an extended experience in the use of the blood treatment and he is an enthusiast on the subject. He makes some very favorable reports of the use of Bovinine in practice; we append some of his reports: Traumatic Bone Necrosis; Case No. xviii; No. 1; Matilda Freyhoff, German, age 26, married; first seen September 27, 1897. Superficial necrosis of the entire first phalanx of left index finger, resulting from a blow causing a periostitis, four weeks ago. The wound was now enlarged by a longitudinal incision, and the necrosed bone cells were removed with the curette. The cavity was thoroughly irrigated with Thiersch's solution, touched up with a 25 per cent solution of pyrozone, and packed with wet Thiersch gauze. After twenty-four hours the dressings were removed, the cavity was cleansed by the bovinine—peroxide process, and packed with bi-sterilized gauze soaked in bovinine. This operation was repeated twice in twenty-four hours, until October 2d; after which it was repeated once in twenty-four hours, until the 7th; at which time the bone was completely siffed and covered with periosteum. The edges of the flesh wound were now freshened and were brought in apposition by strips of rubber plaster. October 10th the patient was discharged cured.

Cartarrhal Case xiv.; No. 4. Eddie Judson, American, single, age 23; case of Dr. B.——. Began treatment September 12, 1897. Chronic rhinitis, which many treatments had failed to relieve. All symptoms of a well-defined case, such as: mucous membrane of the nares very much thickened and of a dark red color; superficial veins dilated and varicosed; several points of ulceration, with considerable loss of structure; profuse, thick, tough, greenish-colored and fetid-smelling secretion. After thoroughly cleansing the passages with bovinine and peroxide of hydrogen, a Thiersch irrigation was employed; after which the points of ulceration were touched up with 25 per cent pyrozone, and the patient was ordered to spray the passages with bovinine and salt water every three hours. October 10th, all symptoms had been removed and the patient was discharged cured.

